# Complete Genome Sequences of Three Listeria monocytogenes Strains from Microgreens Obtained with MinION and MiSeq Sequencing

**DOI:** 10.1128/mra.00277-22

**Published:** 2022-06-06

**Authors:** Sohail Naushad, Amit Mathews, Marc-Olivier Duceppe, Mingsong Kang, Lin Ru Wang, Hongsheng Huang

**Affiliations:** a Ottawa Laboratory–Fallowfield, Canadian Food Inspection Agency, Ottawa, Ontario, Canada; b Greater Toronto Area laboratory, Canadian Food Inspection Agency, Toronto, Ontario, Canada; University of Arizona

## Abstract

Listeria monocytogenes is a Gram-positive, rod-shaped, non-spore-forming bacterium which is an important foodborne bacterial pathogen for human worldwide with 20-30% mortality. Here, we report circular complete genome sequences of three Listeria monocytogenes strains isolated from the samples of microgreens in Canada.

## ANNOUNCEMENT

Listeria monocytogenes is a Gram-positive bacterium found in various environments, animals, and food and is an important foodborne pathogen for humans worldwide with 20 to 30% mortality (1). We report complete genome sequences of L. monocytogenes GTA-L407, GTA-L409, and GTA-L411 strains (Table 1), isolated from three different samples of microgreens taken in Canada in 2018. The strains were isolated by enrichment in UVM broth (30°C, 24 h), followed by isolation on Oxford agar plate (35°C, 48 h) and RAPID'*L.mono* agar plates (35°C for 24 h), followed by the confirmation with Haemolysis on Blood agar plates, motility testing on Trypticase soy agar with yeast extract (30°C, 24 h), and rapid identification with Vitek (bioMérieux, Canada) (2).

**TABLE 1:** Genomic characteristics of the complete genome sequences of 3 Listeria monocytogenes isolated from microgreens

Microgreens type	Strain	No. of Reads		*N*_50_ (bp)	Coverage (x)		Chromosome size (bp)	Predicted CDSs	GC (%)	GenBank no.	SRA nanopore/miseq
		MinION	Miseq	Nanopore reads	MinION	Miseq					
Microgreens-mix	GTA-L407 (also referred as CFIAFB20180112)	135,617	887,969	30,366	398	133	2,865,342	2765	38.06	CP092058	SRR17965221/ SRR17965215
Sunflower	GTA-L409 (also referred as CFIAFB20180114)	59,643	770,315	37,519	291	121	2,879,234	2761	38.01	CP092057	SRR17965220/ SRR17965224
Spring pea	GTA-L411 (also referred as CFIAFB20180116)	41,880	972,281	37,256	209	148	2,890,028	2774	38.01	CP092056	SRR17965219/ SRR17965223

Genomic DNA (gDNA) was extracted from overnight culture (grown from a single colony) in Brain and Heart Infusion medium using Maxwell 16 Cell DNA purification kit (Promega, US) for Illumina sequencing or NanoBind CBB Big DNA kit (Circulomics, US) for Nanopore sequencing. The gDNA was quantified using Qubit (ThermoFisher Scientific, US). Paired-end Illumina library was prepared using Nextera XT Library Preparation kit (Illumina, US) and sequenced for (2 × 300 bp) cycles on Illumina MiSeq. Nanopore sequencing library was generated using the 1D Native barcoding gDNA protocol (EXP-NBD104 and SQK-LSK109) (Oxford Nanopore Technologies, UK), and sequenced using a FLO-MIN106 (R9.4.1) flow cell in MinION MK1C. Basecalling was performed using Super Accuracy mode in Guppy v5.0.11, trimming using Porechop v0.2.3 (3) and filtration using Filtlong v0.2.1 (4). Long reads assembly was performed using Flye v2.7 (5), corrected using Medaka v1.4.4 and polished with Illumina MiSeq reads using a combination of NextPolish v1.4.0, ntEdit v1.3.5 and Polypolish v0.5.0 after trimming/filtering with fastp v0.23.2. Circularity and genome rotation using *dnaA* as the starting point was determined using fixstart plugin from Circlator v1.5.5 (6). Sequencing coverage depth was determined using Minimap2 v2.17 (7) and Samtools v1.13 (8) for long reads and BWA v0.7.17 and Samtools for short reads. Gene prediction and annotation were performed using NCBI Prokaryotic Genome Annotation Pipeline (PGAP)-v6.0 (9).

Antimicrobial resistance (AMR) genes were identified using ResFinder v4.1.5 (10) and RGI v5.2.0 (11). The plasmids were identified by mlplasmids v1.0.0 (12). Prophage sequences were analyzed using PHASTER (13). Default parameters of pipelines were used except otherwise noted (https://github.com/OLF-Bioinformatics/nanopore).

Each strain contains a single chromosome of an average 2,878,201 bp, had 299× (nanopore) and 134× (MiSeq) coverage on average (Table 1), 67 tRNAs and no plasmids and no intact prophage sequences (>90 PHASTER-Score). Each strain contains only *fosX* AMR gene in both ResFinder and RGI analyses. The annotated genome on average had 2,766 coding sequences (CDSs) and 38.08 GC%, similar to 2,889 CDS and 37.88 GC% for L. monocytogenes on average in NCBI (accessed on 2022-03-07).

### Data availability.

The assembled closed genome sequences of the three isolates and their Base-called MinION and MiSeq sequenced reads in fastq format have been deposited in GenBank under the BioProject number PRJNA803486. The accession numbers are provided in Table 1.
